# Supernumerary Teeth in Indian Children: A Survey of 300 Cases

**DOI:** 10.1155/2012/745265

**Published:** 2012-03-20

**Authors:** Amita Sharma, Varun Pratap Singh

**Affiliations:** ^1^Department of Pediatric Dentistry, College of Dental Sciences, BPKIHS, Dharan, Nepal; ^2^Department of Orthodontics, College of Dental Sciences, BPKIHS, Dharan, Nepal

## Abstract

The aim of this investigation was to study children with supernumerary teeth who visited the Department of Pedodontics and Preventive Dentistry, Government Dental College and Hospital, Rohtak, Haryana, India. Only children with supernumerary teeth were included in the study while patients having supernumerary teeth with associated syndromes were excluded. Supernumeraries were detected by clinical and radiographic examination. The results indicated that males were affected more than females with a sex ratio of 2.9 : 1. Single supernumerary tooth was seen in 79% of the patients, 20% had double, and 1% had three or more supernumeraries. Premaxillary supernumeraries accounted for 93.8% of the cases. Conical shaped supernumerary teeth were the most common type (59.7%). Majority of supernumeraries remained unerupted (65%). Fusion of supernumerary tooth with a regular tooth was observed in 4% of the patients. Talon cusp, an associated dental anomaly, was seen in 5% of the cases. Simultaneous hypodontia occurred in 2.3% of patients with supernumeraries.

## 1. Introduction

Supernumerary teeth (hyperdontia) may be defined as extra teeth—more than twenty in the deciduous dentition or more than thirty-two in the permanent dentition [1]. The etiology of supernumerary teeth is not well understood. Several theories have been put forward to explain the anomaly. One theory suggests that supernumeraries are formed as a result of local, conditioned hyperactivity of dental lamina while another theory proposes dichotomy of tooth bud. Heredity plays an important role in the occurrence of supernumerary teeth but does not follow a simple Mendelian pattern. A familial tendency and sex-linked inheritance (males being affected twice as frequently as females) has been demonstrated [2–4].

 Supernumerary teeth occur in 0.3 to 3.8 percent of different populations and appear to be on the rise. Out of these 90 to 98 percent occur in the maxilla with a particular predilection for the premaxilla. Supernumerary teeth may be single or multiple, unilateral or bilateral, erupted or impacted and in one or both jaws. Multiple supernumerary teeth are rare and usually seen in association with cleft lip/palate, Cleidocranial dysplasia, Gardner's syndrome, and so forth [5, 6].

 Supernumerary teeth may be classified according to their form/morphology (supplemental or rudimentary including conical, tuberculate, and molariform types) and location (mesiodens, paramolar, and distomolar). Detection of supernumerary teeth is best achieved with a thorough clinical and radiographic examination. Many complications can be associated with supernumeraries like crowding, delayed eruption, impaction, abnormal diastema, cystic lesions, ectopic eruption, root resorption of adjacent teeth, and so forth. An early diagnosis allows an early intervention, a more favourable prognosis, and minimal complications [7].

 The purpose of this study was to investigate the characteristics of supernumerary teeth among children who reported to our specialty clinic and compare the data with other similar studies.

## 2. Material and Method

A survey was performed on 21,824 patients (11,218 females and 10,606 males) attending the Department of Pedodontics and Preventive Dentistry, Government Dental College and Hospital, Rohtak, Haryana, India over a period of six years. Out of the total population, 300 children with ages ranging from 4 to 14 years were diagnosed with supernumerary teeth in different regions of the dental arches. Reasons for visiting included caries, malocclusion, lack of eruption of permanent teeth, and routine dental check up. The characteristics of supernumerary teeth were noted and diagnosis made during clinical and radiographic examination with help of occlusal, periapical, and panoramic radiographs. The horizontal shift technique was used to determine the sagittal position of the impacted supernumerary teeth. Surgical removal of teeth when and where indicated further confirmed the characteristics of supernumeraries. Patients with syndromes known to be predisposed to supernumerary teeth such as Cleidocranial dysplasia, Gardner's syndrome, clefts of lip, and palate were not included in the study. All the radiographs were reviewed in a negatoscope and interexaminer discrepancies were solved by mutual consensus. The Pearson chi-square test was used to determine potential differences in the distribution of supernumerary teeth when stratified by gender. *P* value of less than 0.01 was considered statistically significant.

## 3. Results

Out of 300 patients, 224 were male, and 76 were female, the sex ratio was 2.9 : 1 (Table 1).

The total number of supernumerary teeth was 385 among the 300 patients. Majority of the patients had single supernumerary tooth (Table 2) which was conical in form (Table 3).

**Figure 1:**
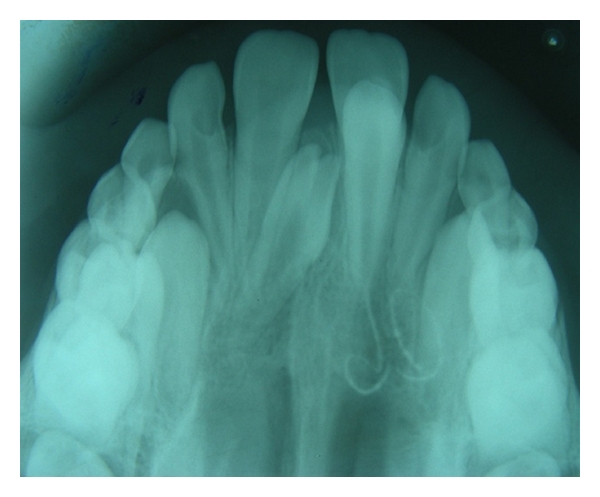
Radiographic appearance of an erupted supplemental 21 along with an unerupted tuberculate supernumerary tooth in maxillary central incisor region.

**Figure 2:**
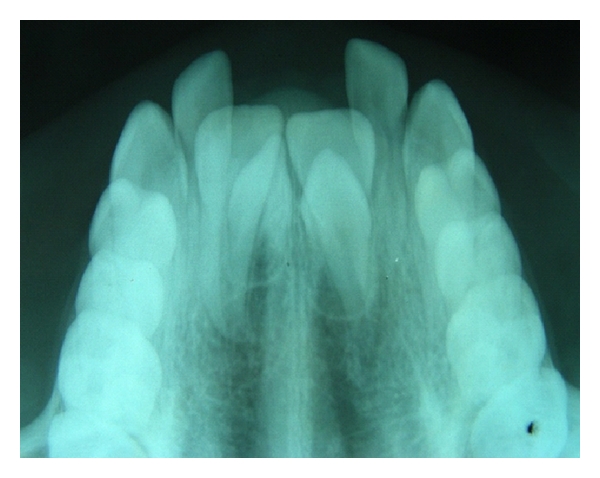
Two unerupted normally oriented conical supernumerary teeth causing failure of eruption of 11 and 21.

**Figure 3:**
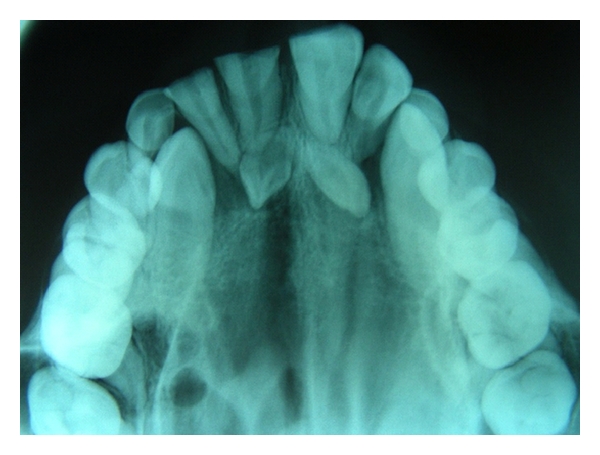
Bilateral unerupted (inverted) conical mesiodentes observed during radiographic examination of fractured anterior teeth.

Supernumerary teeth located in the premaxilla were 361 (93.8%), and out of these, 293 (81.2%) supernumeraries were located in the central incisor region (Figure 1), of which 88 (30.0%) were in midline (mesiodens). Sixty-eight supernumerary teeth were seen in maxillary lateral incisor region (18.8%). The remaining teeth were located in premolar (3.6%), canine (1.0%), and mandibular incisor (1.5%) regions. A large percentage of supernumerary teeth remained unerupted (65%), while 35% were partially or fully erupted (Figures 2 and 3).

**Figure 4:**
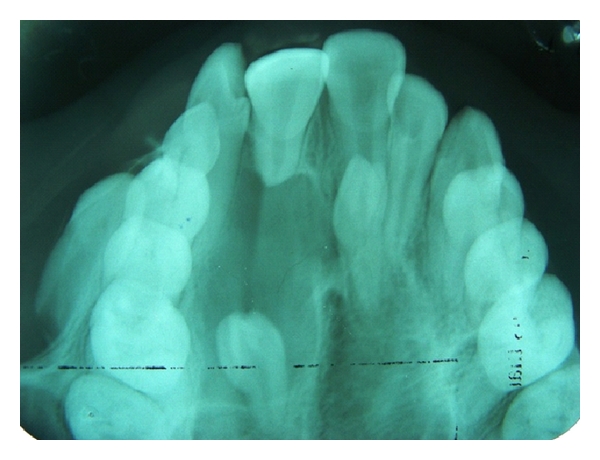
Maxillary occlusal radiograph showing two impacted supernumerary teeth. One on the right side is associated with a radiolucency having sclerotic border suggestive of dentigerous cyst.

Rotation/displacement of adjacent permanent teeth was the most frequently found complication. Three cases of dentigerous cyst associated with supernumerary teeth were detected (Figure 4).

**Figure 5:**
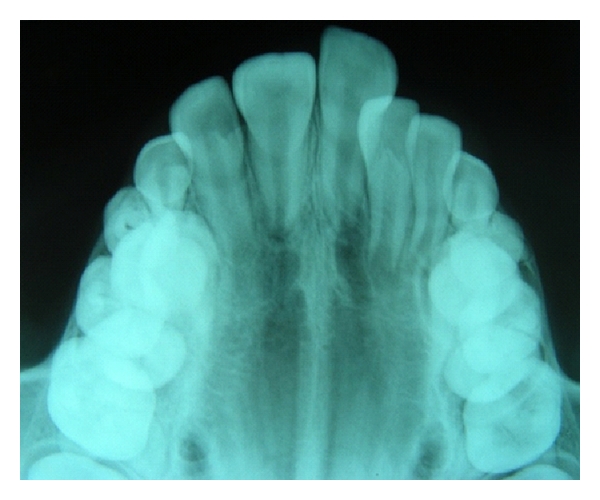
Occlusal radiograph showing talon cusp on 12 and 22; fusion of 22 and supplemental supernumerary 22.

 In 4% of the patients fusion of supernumerary tooth with the adjacent normal tooth occurred. Talon cusp, an associated dental anomaly was seen in 5% of the cases (Figure 5).

**Figure 6:**
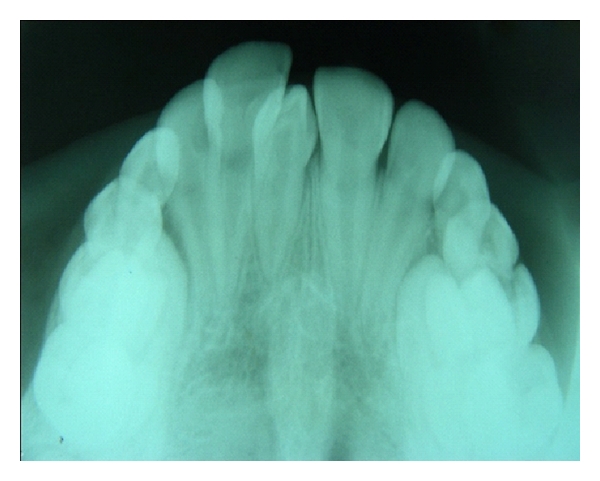
A well-developed mesiodens with talon cusp: a typical superimposed inverted V-shaped radiopaque structure.

Talon cusp on supernumerary teeth was observed in 2% of the patients (Figure 6).

**Figure 7:**
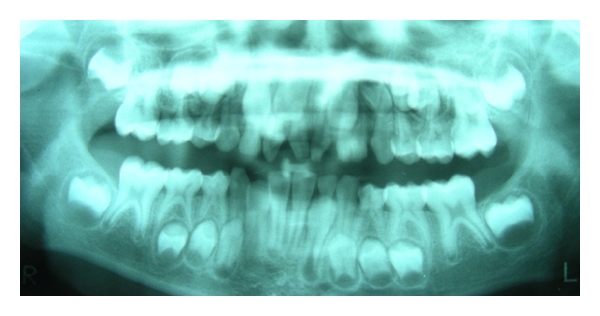
Panoramic radiograph showing bilaterally erupted supplemental mesiodentes; fusion of 61 and 62 and missing 22.

 Seven patients with supernumerary teeth were found having simultaneously congenitally absent teeth (excluding third molars), that is, concomitant hypohyperdontia was observed in 2.3% of the cases (Figure 7).

## 4. Discussion

Table 4 provides an overview of studies done on supernumerary teeth in different populations. Supernumerary teeth most commonly involved the premaxilla which has also been established as the predominant location by others [2, 5, 11–13]. Supernumeraries appeared in a variety of forms (size and shape). Most common was conical, followed by supplemental, then by tuberculate and molariform type (Table 3). Koch et al. reported 56% conical, 12% tubercular, and 11% supplemental and 12% other configurations among their patients [14]. In this study, 35% of the supernumerary teeth were erupted which is higher than that reported in Mckibben's study but comparable to Liu's study [2, 12]. Few authors have reported around 20% of erupted supernumeraries [13, 15].

 The association of talon cusp with other odontogenic anomalies reported in the literature includes peg-shaped lateral incisors, supernumerary teeth, and dens invaginatus, megadont [16, 17]. Very few cases of talon cusp on supernumerary teeth have been reported [18, 19]. Concomitant hypohyperdontia is a rare condition of mixed numeric variation in human dentition. It is usually mentioned as individual case reports in the literature [20]. Its prevalence in orthodontic patients (including third molars) has been reported to be 0.4% [21, 22]. In the present survey on Indian children, a high incidence of other dental anomalies like talon cusp and concomitant hypodontia were seen to be associated with supernumerary teeth while Celikoglu et al. found no such associated dental anomalies in Turkish population [23]. Dentists should take cognizance of associated dental anomalies during the examination for supernumerary teeth so that a comprehensive treatment can be rendered. 

Furthermore as supernumerary teeth are often associated with delayed eruption or impaction of permanent teeth, early removal is recommended to facilitate the spontaneous eruption of impacted permanent teeth [24]. In one interesting study Ashkenazi et al. demonstrated that spontaneous eruption of permanent teeth depends on various variables like apex distance of the impacted tooth relative to its final position, extent of vertical impaction, morphology of supernumerary teeth, angle of impaction relative to midline, and time of surgery. However the authors recommended immediate orthodontic traction at the time of removal of supernumerary teeth [25].

## Figures and Tables

**Table 1:** Distribution of supernumerary teeth by dentition and sex. *Denotes statistically significant values (*P* < 0.01).

Type of dentition	Male	Female	Total	*P* Value
Deciduous	15	5	20	0.002*
Mixed	142	39	181	0.001*
Permanent	67	32	99	0.017

Total	224	76	300	0.002*

**Table 2:** Distribution of supernumerary teeth by number per patient.

Number of teeth per patient	Number of patients	Percentage of supernumerary teeth
One	237	79.0
Two	60	20.0
Three or more	03	01.0

Total	300	100.0

**Table 3:** Type of supernumerary tooth.

Type	Number	Percentage
Supplemental (Eumorphic)	70	18.2
Rudimentary (Dysmorphic): Conical	230	59.7
Tuberculate	55	14.3
Molariform	30	07.8

Total	385	100.0

**Table 4:** Summary of various studies carried out on supernumerary teeth in different populations.

Authors	Sample size	Country	Age	Method	Types of supernumerary included	Male : Female
Present study	300 children diagnosed with supernumerary teeth	India	4–14 Yeras	Clinical Examination and radiographs	All	2.9 : 1
Tyrologou et al. (2005) [8]	97 children with diagnosed mesiodens	Sweden	3–15 years	Clinical examination and radiographs	Mesiodens	2 : 1
Huang et al. (1992) [9]	152 children with diagnosed supernumerary teeth	Jordan	5–15 years	Clinical examination and radiographs	All	2.2 : 1
Liu (1995) [2]	112 children with diagnosed supernumerary teeth in the premaxillary regions	Taiwan	4–14 years	Clinical examination and radiographs	In the Premaxillary region.	2.8 : 1
von Arx (1992) [10]	90 patients with anterior maxillary supernumerary	Switzerland	6–10 years	Clinical examination and radiographs	In the Anterior Maxillary region	2.6 : 1
